# Comparative Evaluation of Six SARS-CoV-2 Real-Time RT-PCR Diagnostic Approaches Shows Substantial Genomic Variant–Dependent Intra- and Inter-Test Variability, Poor Interchangeability of Cycle Threshold and Complementary Turn-Around Times

**DOI:** 10.3390/pathogens11040462

**Published:** 2022-04-12

**Authors:** Rok Kogoj, Misa Korva, Nataša Knap, Katarina Resman Rus, Patricija Pozvek, Tatjana Avšič-Županc, Mario Poljak

**Affiliations:** Institute of Microbiology and Immunology, Faculty of Medicine, University of Ljubljana, Zaloška Cesta 4, 1000 Ljubljana, Slovenia; rok.kogoj@mf.uni-lj.si (R.K.); misa.korva@mf.uni-lj.si (M.K.); natasa.knap@mf.uni-lj.si (N.K.); katarina.resman@mf.uni-lj.si (K.R.R.); patricija.pozvek@mf.uni-lj.si (P.P.)

**Keywords:** severe acute respiratory syndrome coronavirus 2 (SARS-CoV-2), coronavirus disease 19 (COVID-19), genomic variant, real-time RT-PCR, Ct value, turn-around time

## Abstract

Several professional societies advise against using real-time Reverse-Transcription PCR (rtRT-PCR) cycle threshold (Ct) values to guide clinical decisions. We comparatively assessed the variability of Ct values generated by six diagnostic approaches by testing serial dilutions of well-characterized isolates of 10 clinically most relevant SARS-CoV-2 genomic variants: Alpha, Beta, Gamma, Delta, Eta, Iota, Omicron, A.27, B.1.258.17, and B.1 with D614G mutation. Comparison of three fully automated rtRT-PCR analyzers and a reference manual rtRT-PCR assay using RNA isolated with three different nucleic acid isolation instruments showed substantial inter-variant intra-test and intra-variant inter-test variability. Ct value differences were dependent on both the rtRT-PCR platform and SARS-CoV-2 genomic variant. Differences ranging from 2.0 to 8.4 Ct values were observed when testing equal concentrations of different SARS-CoV-2 variants. Results confirm that Ct values are an unreliable surrogate for viral load and should not be used as a proxy of infectivity and transmissibility, especially when different rtRT-PCR assays are used in parallel and multiple SARS-CoV-2 variants are circulating. A detailed turn-around time (TAT) comparative assessment showed substantially different TATs, but parallel use of different diagnostic approaches was beneficial and complementary, allowing release of results for more than 81% of non-priority samples within 8 h after admission.

## 1. Introduction

Following the initial emergence of Severe Acute Respiratory Syndrome Coronavirus 2 (SARS-CoV-2) and its subsequent global pandemic spread, a range of molecular SARS-CoV-2 RNA assays, both in-house and commercial, were rapidly developed following the real-time Reverse-Transcription PCR (rtRT-PCR) protocols summary by the WHO (https://www.who.int/docs/default-source/coronaviruse/whoinhouseassays.pdf, accessed on 22 March 2022). Consequently, a substantial number of SARS-CoV-2 RNA assays received In Vitro Diagnostics Emergency Use Authorization (EUA) approval from the US Food and Drug Administration (FDA) (https://www.fda.gov/medical-devices/coronavirus-disease-2019-covid-19-emergency-use-authorizations-medical-devices/in-vitro-diagnostics-euas-molecular-diagnostic-tests-sars-cov-2, accessed on 22 March 2022). Due to the unprecedented need for SARS-CoV-2 testing, significant supply problems and a shortage of sample devices, instruments, reagents, and consumables soon arose and are ongoing [1,2]. To keep up with the sheer volume of test requests, several laboratories were forced to implement multiple SARS-CoV-2 molecular assays, choosing from different manufacturers and diverse assay designs [3,4], resulting in potentially clinically relevant inter-laboratory assay-to-assay variations and poor result interchangeability.

Real-time RT-PCR is still considered the SARS-CoV-2 reference laboratory diagnostic standard [5,6] due to its high analytical sensitivity and specificity, multiplexing ability, acceptable turn-around time (TAT), and scalability [7]. Another advantage of rtRT-PCR over other amplification methods is its quantification capability when determination of viral load is clinically relevant [8]. To help healthcare professionals better manage the COVID-19 pandemic, significant efforts are being made to correlate rtRT-PCR cycle threshold (Ct) values with disease severity [8,9,10], with viral shedding as a predictor of active infection and transmissibility [11], with duration of infectivity and transmissibility [12], for quarantine/isolation duration and termination [13], for discharge of patients from wards [14], for assessing potential cases of reinfection [15], and as an early indicator of a SARS-CoV-2 surge [16]. However, definitive data to support the predictive value of Ct values in these situations are lacking and, due to the myriad of analytical and clinical factors known to impact Ct values, several professional societies and authorities—including the Infectious Disease Society of America (IDSA) together with the Association for Molecular Pathology (AMP); https://www.idsociety.org/globalassets/idsa/public-health/covid-19/idsa-amp-statement.pdf (accessed on 22 March 2022), American Association for Clinical Chemistry (AACC); https://www.aacc.org/cln/articles/2021/december/how-to-say-no-to-reporting-ct-values (accessed on 22 March 2022), Public Health England (PHE); https://assets.publishing.service.gov.uk/government/uploads/system/uploads/attachment_data/file/926410/Understanding_Cycle_Threshold__Ct__in_SARS-CoV-2_RT-PCR_.pdf (accessed on 22 March 2022), Indian Council of Medical Research (ICMR); https://www.icmr.gov.in/pdf/covid/techdoc/Advisory_on_correlation_of_COVID_severity_with_Ct_values.pdf (accessed on 22 March 2022), Association of Public Health Laboratories (APHL); https://www.aphl.org/programs/preparedness/Crisis-Management/Documents/APHL-COVID19-Ct-Values.pdf (accessed on 22 March 2022), and Government of Canada; https://www.canada.ca/en/public-health/services/diseases/2019-novel-coronavirus-infection/guidance-documents/polymerase-chain-reaction-cycle-threshold-values-testing.html#a5 (accessed on 22 March 2022)—advise caution when applying Ct values for these indications and advise against the routine use of Ct values to guide clinical decisions. Consequently, many laboratories, including the Laboratory for COVID-19 Diagnostics at the Institute of Microbiology and Immunology (IMI), Faculty of Medicine, University of Ljubljana, do not routinely include Ct values in laboratory reports, opting instead to provide them to responsible clinicians on a case-by-case basis.

The main aim of this study was to comparatively assess the variability of Ct values generated by six previously thoroughly evaluated rtRT-PCR assays and platforms [17,18,19] by testing serial dilutions of well-characterized SARS-CoV-2 isolates of the clinically most relevant genomic variants (Alpha, Beta, Gamma, Delta, Eta, Iota, Omicron, A.27, B.1.258.17, and B.1 with D614G mutation). Thus, the performance of three fully automated rtRT-PCR analyzers—cobas 6800 (Roche, Basel, Switzerland), STARLet (Seegene, Seoul, South Korea), and Alinity_m (Abbott, Chicago, IL, USA)—and three diagnostic approaches using different instruments for automated nucleic acid isolation: Maelstrom 9600 (TANbead, Taoyuan, Taiwan), MagNA Pure 96 (Roche), and NX-48 (Genolution, Seoul, South Korea) followed by reference manual rtRT-PCR LightMix Wuhan CoV kit (TIB MOLBIOL, Berlin, Germany), was compared. In addition, the impact of 10 selected SARS-CoV-2 genomic variants on the performance of each of the diagnostic approaches was assessed. Finally, TATs of the six diagnostic approaches in a real-life laboratory setting were meticulously measured and comparatively evaluated.

## 2. Results

### 2.1. Inter-Variant, Intra-Test Ct Value Variability of Six SARS-CoV-2 Diagnostic Approaches

The results of testing in triplicate of six different concentrations of 10 well-characterized samples containing different SARS-CoV-2 genomic variants using six diagnostic approaches are shown in Table 1. None of the dilutions were SARS-CoV-2 RNA–negative for any of the target genes when tested using three fully automated rtRT-PCR analyzers. Inter-variant Ct values SDs of about one log were observed throughout all concentrations tested for all targets by all analyzers with the exception of STARLet’s target gene N. For this target, inter-variant Ct value SDs between 2.2 and 2.8 log were observed at respective concentrations (Table 1). For STARLet’s target gene N, mean Ct values at all concentrations tested were also significantly higher (*p* < 0.001) for Beta, Eta, and Iota variants in comparison to other SARS-CoV-2 genomic variants tested (Supplemental Table S1). No such deviations were observed for any of the targets in all assays tested regardless of SARS-CoV-2 genomic variant included in this study. Similarly, none of the dilutions were SARS-CoV-2 RNA–negative for any of the target genes when tested by LightMix after nucleic acid isolation using three different analyzers. The SDs of LightMix’s Ct values in samples processed by three different nucleic acid isolation systems were also about 1 log across all serial dilutions created, which is comparable to the data obtained by fully automated integrated rtRT-PCR analyzers (Table 1).

### 2.2. Intra-Variant, Inter-Test rtRT-PCR Efficiency Variability of Six SARS-CoV-2 Diagnostic Approaches

Different numbers of rtRT-PCR targets, target gene regions, or even a combination of targets are used in the diagnostic approaches evaluated. Therefore, rtRT-PCR efficiency was chosen rather than Ct values for comparison of results obtained by six diagnostic approaches related to respective SARS-CoV-2 genomic variants. As presented in Table 2, the comparative assessment showed that, regardless of the SARS-CoV-2 variant, all six diagnostic approaches performed with rtRT-PCR efficiencies between 80.1% and 106.8%. However, rtRT-PCR efficiencies for the same genomic variant were not consistent between assays. Regardless of the SARS-CoV-2 variant tested, cobas showed the most consistent rtRT-PCR efficiency, ranging from 98.8% to 105.0% and from 95.8% to 106.8% for Target 1 (ORF1ab) and Target 2 (E gene), respectively (Table 2).

**Figure 1:**
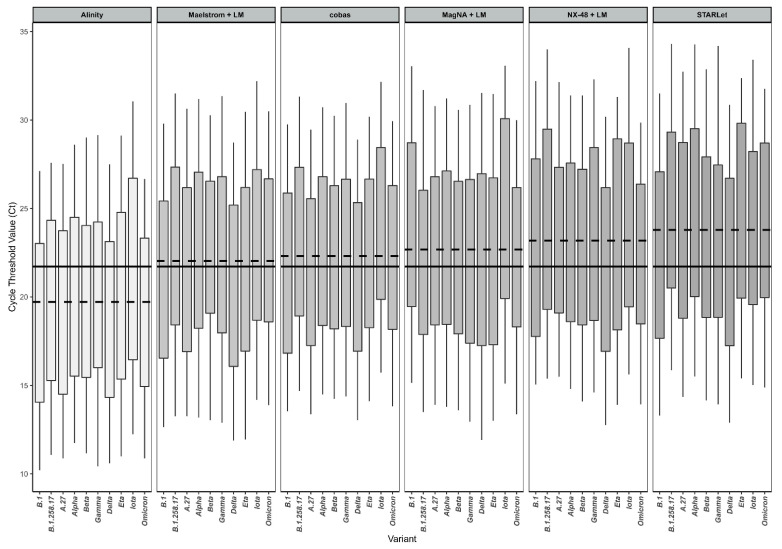
Distribution of RdRP Ct values generated by six diagnostic approaches evaluated across 10 SARS-CoV-2 genomic variants. Dashed lines represent the mean Ct value obtained with each respective diagnostic approach across all 10 genomic variants tested. The full line represents the overall mean of Ct values obtained in all experiments. The boxes represent interquartile range (difference between first and third quartile, IQR), and whiskers represent the remaining quartile of measures (12.5% on each side). The lower and upper limits of the whiskers represent the highest and lowest Ct values measured.

Overall, the targets in the respective assays were amplified with a mean inter-test rtRT-PCR efficiency of at least 91% for each genomic variant tested, with the exception of the Gamma (P.1) variant, with a mean inter-test rtRT-PCR efficiency of 88.5% (Table 2). Cobas had the best rtRT-PCR efficiency (100.1% Target 1; 96.8% Target 2) for detection of the Gamma variant, whereas in all other diagnostic approaches rtRT-PCR efficiency for this genomic variant did not reach the 90% threshold, the lowest being for STARLet gene N (80.6%) (Table 2). Direct comparison of Ct values between different diagnostic approaches is hampered because each assay/analyzer used different targets, chemistry, and design, but the RdRP gene was represented as a target in all six approaches chosen. Thus, detailed head-to-head Ct comparison between six diagnostic approaches was performed for the RdRP gene only (Table 3 and Figure 1). The results show substantial diagnostic approach–dependent differences in mean Ct values across all dilutions tested, looking at genomic variants separately. At respective concentrations, the differences between the platform with the maximum mean Ct value and the platform with the minimum mean Ct value span from 2.4 to 7.1. If all genomic variants are considered together, these differences are even greater: between 5.7 and 7.6 (Table 3). Figure 1 shows the distribution of RdRP mean Ct values generated by six diagnostic approaches evaluated for 10 SARS-CoV-2 genomic variants tested. It reveals that, when testing the same concentrations of either genomic variant, the resulting Ct range varies greatly and is dependent on the diagnostic approach and genomic variant. Assessment of systemic error, robustness of the systems used, and pipetting accuracy are presented in Supplemental Figure S1.

### 2.3. Turn-Around Times of Six SARS-CoV-2 Diagnostic Approaches

**Figure 2:**
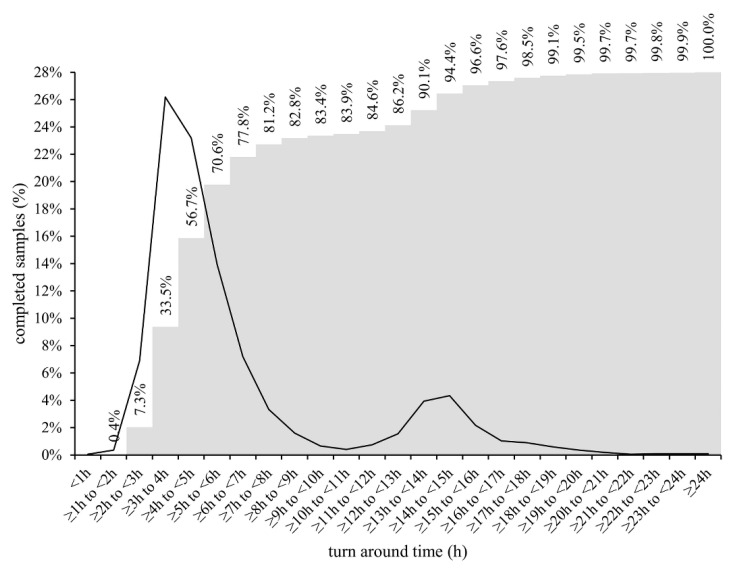
Percentage of regular non-priority samples with completed test results at respective time intervals after admission to the laboratory (primary axis = black line) and cumulative number of samples with completed test results at respective time intervals after admission to the laboratory (secondary axis = gray columns) regardless of the diagnostic approach used.

**Figure 3:**
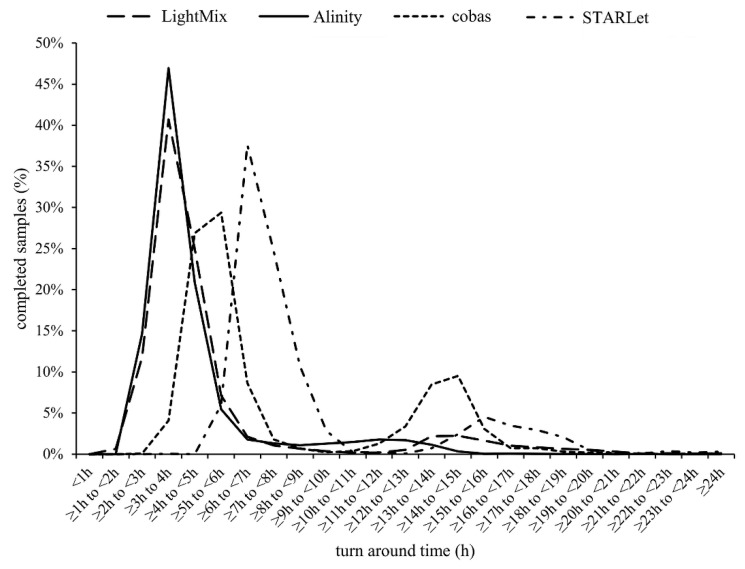
Percentage of regular non-priority samples with completed test results at respective time intervals after admission to the laboratory by diagnostic approach used.

TATs were comparatively assessed during the period when all six diagnostic approaches were used in parallel. From March 2020 to the end of May 2021, a total of 504,173 samples for SARS-CoV-2 RNA testing were received at IMI. Of these, 96.1% (484,336/504,173) were processed as regular samples. The remaining 3.9% (19,837/504,173) were ordered as high-priority samples (STAT) and were processed either by XpertXpress SARS-CoV-2 (Cepheid, Sunnyvale, CA, USA) or cobas LIAT (Roche). However, due to the frequent supply shortage of cartridges/assay tubes needed for two ultra-fast rtRT-PCRs, 29.9% (5933/19,837) of STAT samples were processed with LightMix or Alinity, amounting to a total of 490,269 samples included in the TAT analysis. As shown in Figure 2, cumulatively, 0.4% (1961/490,269), 7.3% (35,790/490,269), 33.5% (164,240/490,269), 56.7% (277,983/490,269), 70.6% (346,130/490,269), 77.8% (381,429/490,269), and 81.2% (398,098/490,269) of samples were processed in the first 2, 3, 4, 5, 6, 7, and 8 h after admission to the laboratory, respectively. Most results were obtained during the major and minor time peak period at 3 to 7 h and 12 to 17 h after admission of samples to the laboratory, respectively (Figure 2), with marked differences between diagnostic approaches (Figure 3). Alinity and LightMix were able to provide final results in 40.7% (29,608/63,078) and 46.9% (109,201/232,836) of samples, respectively, within 3 to 4 h after admission to the laboratory. Cobas completed 26.9% (41,088/152,909) of samples within 4 to 5 h and 29.4% (44,893/152,909) of samples within 5 to 6 h, and STARLet 37.6% (15,589/41,446) of samples within 6 to 7 h after admission to the laboratory (Figure 3).

## 3. Discussion

In the past 2 years, molecular laboratories around the world were forced to unprecedentedly increase their capacity for rtRT-PCR testing to meet extraordinary demands for SARS-CoV-2 RNA testing from authorities and healthcare facilities. In addition to high-volume testing, laboratories were challenged daily by several interpretation issues, one of the most frequent being applying rtRT-PCR Ct values for better management of COVID-19 patients. Predicting and determining the duration of infectivity and transmissibility is indeed a very important aspect in management of COVID-19 patients as well as exposed individuals to allow timely hospital discharge, and as short a quarantine and isolation duration as possible and their appropriate termination. Although the quantification ability of rtRT-PCR is indispensable in management of several important infectious diseases, the use of Ct values in management of COVID-19 is still controversial [20]. Namely, due to numerous analytical and clinical factors known to impact Ct values in general, the non-standardized nature of sample collection using a nasopharyngeal swab, and the fact that patients that have recovered from COVID-19 can remain rtRT-PCR–positive for a prolonged period of time [12,21], there is a consensus that the mere presence of SARS-CoV-2 RNA does not necessarily reflect the infectivity and transmissibility of a patient [22,23]. Despite all these uncertainties and the almost uniform recommendation of professional societies and some authorities and findings by researchers [24] against the routine use of Ct values to guide clinical decisions, a general concept that classifies patients with a Ct value of ≥30 as non-infectious unfortunately seems to have prevailed in peer-reviewed literature [25,26].

To the best of our knowledge, no comprehensive comparison of Ct values and rtRT-PCR efficiencies generated by several rtRT-PCR platforms and various nucleic acid isolation methods across a range of the clinically most relevant SARS-CoV-2 genomic variants has been performed yet. For this study, all variants of concern (VOC) and variants of interest (VOI) detected in the central European region until the end of 2021 were selected for analysis. In addition, variant B.1.258.17 as the main lineage during the second wave, variant A.27 as a rare variant, with mutation N501Y, and variant B.1 (with D614G) as the first main lineage, which caused a substantial part of the first wave of infections in Europe, were included. All 10 genomic variants were tested in triplicate using six selected diagnostic approaches at concentrations ranging from 1 × 10^4^ PFU/mL to 1 × 10^−1^ PFU/mL. This study clearly showed substantial differences in Ct values generated by six selected diagnostic approaches with respect to the SARS-CoV-2 genomic variant, even without taking into account sample quality variability. Differences ranging from 2.0 to 8.4 Ct values were observed for the same concentration of different SARS-CoV-2 genomic variants, clearly showing a relevant influence of genomic variant on the detection capability of rtRT-PCRs. Although larger Ct variability was initially expected for manual diagnostic approaches over fully automated analyzers, it turned out that SD values for respective concentrations were quite similar between manual and automated approaches, except when targeting STARLet’s N gene. In addition, for STARLet’s N gene, significantly higher Ct values (*p* < 0.001) for the Beta (B.1.351), Eta (B.1.525), and Iota (B.1.526) genomic variants were found at all concentrations tested compared to other genomic variants included in the study. Similar deviations for the SARS-CoV-2 Alpha genomic variant were previously observed with the SARS-CoV-2/FluA/FluB/RSV assay [27,28,29]. There is reason to believe that these genomic variants most likely have mutation(s) in the N gene at primers and/or probe binding site(s) that affect the amplification efficacy and/or intensity of the probe signal, resulting in higher Ct values. In this study, such N-gene Ct value deviations were not observed for the Alpha genomic variant using the SARS-CoV-2 specific Allplex assay. With other diagnostic approaches included in this study such deviations were not observed for any of the rtRT-PCR targets regardless of SARS-CoV-2 genomic variant tested. These results clearly indicate that Ct values as a surrogate for viral load are unreliable and should not be used as an indicator of infectivity and transmissibility, especially when different rtRT-PCR assays are used in the laboratory in parallel and multiple SARS-CoV-2 genomic variants are circulating in a population. This observation is supported by the findings of Arons et al. [30] and Scola et al. [14], who have shown that 25% and even 50% of clinical samples with Ct values above 30 can be cultured and could be potentially infectious. In addition, the results of recent external quality assessment showed that Ct values reported by laboratories can vary by more than ±4 (up to 18) depending on the nucleic acid extraction protocol and rtRT-PCR platform used [31]. Furthermore, the results of this study showed that some SARS-CoV-2 genomic variants are not amplified with equal rtRT-PCR efficiency using different rtRT-PCR assays. Although all targets in the diagnostic approaches assessed were amplified with rtRT-PCR efficiencies between 80 and 120%, which is generally considered acceptable, rtRT-PCR efficiencies for some SARS-CoV-2 genomic variants showed a 14.8 to 24.1% difference between diagnostic approaches with potential patient management consequences. Such differences are best recognizable when mean Ct values for the RdRP gene, which is a common target for all six diagnostic approaches, are compared head-to-head. The RdRP gene mean Ct values varied from 2.4 to 7.1 across diagnostic approaches tested when the genomic variants are considered individually, and when the results are calculated independent of genomic variant, the differences for given concentrations ranged from 5.7 to 7.6 at respective concentrations across the diagnostic approaches tested. This clearly indicates that the use of the Ct value 30 as a simple cutoff for infectivity and transmissibility is inappropriate and that the Ct values measured are dependent on the diagnostic platform and genomic variant. However, quantification precision can be significantly improved by using standardized quantitative rtRT-PCR or droplet digital PCR [32].

Several strategies have been employed by laboratories worldwide to meet the high COVID-19 testing demand, including pooling of samples [33,34,35], multiplexing [36,37,38], and omission or simplification of nucleic acid isolation [39,40], all with expected tradeoffs in overall analytical sensitivity. In many laboratories, including our laboratory, requested diagnostic scaling up could only be achieved by using several molecular platforms in parallel, ranging from fully automated integrated sample-to-result analyzers such as cobas or Alinity to different semi-automated or manual approaches. Although such a strategy was proven to be beneficial timewise, the use of diverse diagnostic approaches in parallel undeniably led to potentially clinically relevant intra-laboratory assay-to-assay variations and poor result interchangeability, as shown in this study.

However, on the positive side, having diverse diagnostic approaches running in parallel allowed us to meticulously measure and comparatively evaluate TATs of different diagnostic approaches in a real-life laboratory setting. Overall, during three SARS-CoV-2 waves, a combination of six automated and manual diagnostic approaches made possible the release of final results for more than 81% of routine non-priority samples within 8 h after admission to the laboratory. A detailed TAT analysis showed that most results were released in two peak time periods, the major one being within the first 8 h after admission (Figure 2). This first peak time period was composed of several sub-peaks (Figure 3), the first containing samples processed by LightMix and Alinity, followed by cobas and finally STARLet. Both Alinity and LightMix were found to be comparable in terms of TAT and the fastest of the diagnostic approaches comparatively assessed, with TAT in 3 to 4 h. This observation is not surprising because Alinity takes 140 min to process a batch of the first 12 samples, adding another 12 samples every 16 min. Similarly, LightMix, in conjunction with the slowest of the automated nucleic acid isolation approaches assessed (MagNA), requires up to 160 min for 96 samples. Cobas follows with mean TAT of 4 to 6 h, processing samples in batches of up to 94 in 180 min. The slowest of the diagnostic approaches evaluated was STARLet, which took 6 to 7 h to process most of the samples because it had the longest processing time for batches of 94 samples (250 min). Although having substantially different individual TATs, a parallel use of six diagnostic approaches was found to be quite complementary in a real-life laboratory setting, allowing day-to-day flexibility, testing of up to 8000 samples in 24 h without significant prolongation of TAT, and providing a valuable back-up solution in cases of technical issues, failures, and temporary reagent shortages. Other possibilities to shorten TAT are available such as ultrafast PCR [41,42] and isothermal assays [43,44] however, implementation of such methods in parallel to rtRT-PCR may bring even further complexity and complications when a request for Ct value is made.

There are some important limitations of this study. The input volume of samples differed slightly across the diagnostic approaches evaluated, both automated and manual, but it was dictated by manufacturers’ instructions. Unfortunately, the elution volumes during RNA extraction and the volumes used for subsequent rtRT-PCR in the automated analyzers are not disclosed by the manufacturers. The sample input volumes as well as elution volumes for the three automated nucleic acid isolation platforms were also slightly different. In addition, virus quantification by PFU is associated with some degree of inaccuracy due to the method design, which cannot be fully avoided [45]. Finally, only the clinically most relevant VOCs, VOIs, and major lineages circulating in central Europe until the end of 2021 were tested. Although the factors listed may introduce some biases and variability in the Ct values generated, we remain confident that the differences in Ct values and rtRT-PCR efficiencies identified in this comparative study are mainly driven by differences in the assays’ design and are genomic variant–dependent.

## 4. Conclusions

In summary, testing serial dilutions of well-characterized SARS-CoV-2 isolates of a range of the clinically most relevant SARS-CoV-2 genomic variants using six previously thoroughly evaluated diagnostic approaches showed substantial inter-variant intra-test as well as intra-variant inter-test variability. The differences obtained in Ct values were dependent on both the rtRT-PCR platform and SARS-CoV-2 genomic variant. This study further supports recommendations against the routine use of Ct values to guide clinical decisions and reconfirms the need for close monitoring of new emerging SARS-CoV-2 genomic variants and their potential impact on the performance of rtRT-PCR assays. A detailed TAT analysis showed that, despite substantially different individual TATs in a real-life laboratory setting, the parallel use of several diagnostic approaches was beneficial and complementary and made possible the release of final results for more than 81% of routine non-priority samples within 8 h after admission to the laboratory during three SARS-CoV-2 waves.

## 5. Materials and Methods

### 5.1. SARS-CoV-2 Genomic Variant Selection

Since March 2020, complete SARS-CoV-2 genomes have been routinely sequenced at IMI with next-generation sequencing (NGS) using residual nasopharyngeal swab samples of COVID-19–positive patients to actively monitor the epidemiology, circulation, and emergence of SARS-CoV-2 variants in the country. All fully characterized samples have been stored at −70 °C and whole genome sequences uploaded to the GISAID database (https://www.gisaid.org, (accessed on 22 March 2022)). Selected samples with Ct values below 25 have been additionally cultured on Vero E6 cells and quantified by TCID50 as described previously [46]. The following 10 SARS-CoV-2 variants were selected for this study: Alpha (B.1.1.7), Beta (B.1.351), Gamma (P.1), Delta (B.1.617.2), Eta (B.1.525), Iota (B.1.526), Omicron (B.1.1.529) A.27, B.1.258.17, and B.1 with D614G mutation, all of which are deposited in the EVA-GLOBAL Virus Archive under the following reference numbers (Ref-SKU): 005V-04053, 005V-04107, 005V-04248, 005V-04249, 005V-04109, 005V-04401, 005V-04479, 005V-04144, 005V-04394, and 005V-03961. The genomes can be downloaded from GISAID under the following references: EPI_ISL_877453, EPI_ISL_1118868, EPI_ISL_1240606, EPI_ISL_1935543, EPI_ISL_1181833, EPI_ISL_5305342, EPI_ISL_9007956, EPI_ISL_1668566, EPI_ISL_1668577, and EPI_ISL_635205.

### 5.2. SARS-CoV-2 rtRT-PCRs

Six different diagnostic approaches were comparatively evaluated: three fully automated and integrated rtRT-PCR analyzers and a reference manual rtRT-PCR assay using RNA isolated with three different instruments for automated nucleic acid isolation. All testing was performed strictly following the respective manufacturers’ instructions. Briefly, logarithmic dilutions of nine samples containing the SARS-CoV-2 genomic variants listed above were prepared in fresh RPMI medium (Sigma Aldrich, St. Louis, MO) to obtain 1 × 10^4^/1 × 10^3^/1 × 10^2^/1 × 10^1^/1 × 10^0^/1 × 10^−1^ PFU/mL. The dilutions were distributed in aliquots of appropriate volume and tested in triplicate with cobas SARS-CoV-2 Test (Roche) on a cobas 6800 analyzer (cobas), Allplex SARS-CoV-2 Assay (Seegene) on a STARLet analyzer (STARLet), and SARS-CoV-2 Kit (Abbott) on an Alinity_m analyzer (Alinity). Similarly, for manual LightMix Wuhan CoV kits (LightMix), nucleic acids were isolated from triplicates of appropriate sample volume for each automated nucleic acid isolation instrument: 190 µL for NX-48, 200 µL for MagNA Pure 96, and 300 µL for Maelstrom 9600. Nucleic acids were eluted in volumes as recommended by the manufacturers. Isolated RNA and reagents for rtRT-PCRs were automatically pipetted into 384-well PCR plates (Applied Biosystems, Foster City, CA, USA) using a Janus G3 pipetting robot (Perkin Elmer, Waltham, MA, USA) dispensing 5 µL of RNA eluate and 7.5 µL of rtRT-PCR master mix per well. Two separate master mixes were prepared from 1 × TaqMan FastVirus 1-Step Master Mix (Applied Biosystems) and each Wuhan SARS-CoV-2 E gene and Wuhan SARS-CoV-2 RdRP gene kits. Real-time RT-PCR amplification and detection were performed using a QuantStudio7Pro instrument (Applied Biosystems). Ct values generated by six diagnostic approaches were collected and, after computing mean Ct values for each concentration tested, used for calculation of respective rtRT-PCR efficiencies obtained from the slopes of linear regression lines across dilutions tested using the following equation: Efficiency (%) = ((10e (−1/slope)) − 1) × 100). PCR efficiency of 100% means that in each PCR cycle the amount of target is doubled. Statistical calculations and graph plotting were performed using Excel 2016 version 16.0.5188.1000 (Microsoft, Redmond, MA, USA), Prism7 version 7.04 (GraphPad, San Diego, CA, USA), and R software version 4.1.2 (The R Foundation for Statistical computing, Vienna, Austria).

### 5.3. Turn-Around Time of Six Diagnostic Approaches

Data for laboratory admission time and time-to-result reporting were analyzed from March 2020 until the end of May 2021 and used to calculate the total TAT for each of the six diagnostic approaches. Only routinely processed samples tested with cobas, STARLet, Alinity, and LightMix were included in the TAT analysis, and fast-track prioritized samples were not considered.

## Figures and Tables

**Table 1:** Inter-variant mean Ct values with standard deviations (SD), coefficient of variation (CV), and individual variant lowest mean/highest mean Ct at respective concentration points generated by specific PCR targets in all rtRT-PCR diagnostic approaches assessed (Alinity, cobas, STARLet, and LightMix) when testing in triplicate six different concentrations of 10 well-characterized isolates containing different SARS-CoV-2 genomic variants.

Concentration	1 × 10^4^ [PFU/mL]			1 × 10^3^ [PFU/mL]			1 × 10^2^ [PFU/mL]			1 × 10^1^ [PFU/mL]			1 × 10^0^ [PFU/mL]			1 × 10^−1^ [PFU/mL]		
Alinity	N/R †			N/R †			N/R †			N/R †			N/R †			N/R †		
inter-variant mean Ct ± SD	11.0 ± 0.6			14.3 ± 0.8			18.0 ± 0.8			21.7 ± 0.8			25.0 ± 1.2			28.3 ± 1.3		
var. ^+^ lowest mean Ct var. ^+^ highest mean Ct	10.2 12.2			13.0 15.3			17.1 19.9			20.6 23.4			23.8 27.8			26.7 31.1		
inter-variant CV [%]	5.5			5.6			4.4			3.7			4.8			4.6		
cobas	ORF ‡	E		ORF ‡	E		ORF ‡	E		ORF ‡	E		ORF ‡	E		ORF ‡	E	
inter-variant mean Ct ± SD	14.1 ± 0.8	14.4 ± 0.9		17.2 ± 0.9	17.6 ± 1.0		20.7 ± 1.0	21.2 ± 1.0		24.1 ± 0.9	24.6 ± 0.9		27.3 ± 0.9	27.8 ± 1.0		30.4 ± 1.0	31.0 ± 1.2	
var. ^+^ lowest mean Ct var. ^+^ highest mean Ct	13.0 15.7	13.0 16.0		16.0 19.0	15.7 19.4		19.3 22.6	19.4 23.0		22.9 25.9	23.1 26.3		26.2 29.3	26.2 29.8		28.9 32.2	28.9 33.1	
inter-variant CV [%]	5.7	6.3		5.2	5.7		4.8	4.7		3.7	3.7		3.3	3.6		3.3	3.9	
STARLet	E	R †/S	N	E	R †/S	N	E	R †/S	N	E	R †/S	N	E	R †/S	N	E	R †/S	N
inter-variant mean Ct ± SD	15.3 ± 0.8	14.5 ± 1.0	15.6 ± 2.8	18.5 ± 1.2	18.1 ± 1.1	19.4 ± 2.5	22.3 ± 1.3	22.2 ± 1.1	23.2 ± 2.4	25.8 ± 1.2	25.8 ± 1.0	26.2 ± 2.2	28.9 ± 1.1	29.2 ± 1.1	29.8 ± 2.4	32.4 ± 1.5	32.8 ± 1.2	33.7 ± 2.7
var. ^+^ lowest mean Ct var. ^+^ highest mean Ct	13.4 16.4	12.9 15.9	11.3 19.7	15.9 19.7	16.1 19.4	16.0 23.4	19.5 23.7	20.6 23.7	19.7 26.9	23.5 26.8	24.3 27.0	23.2 30.0	26.5 30.2	27.5 30.8	26.6 33.1	29.4 34.0	30.9 34.3	29.9 37.9
inter-variant CV [%]	5.2	6.9	17.9	6.5	6.1	12.9	5.8	5.0	10.3	4.7	3.9	8.4	3.8	3.8	8.1	4.6	3.7	8.0
Maelstrom + LM *	E	R †		E	R †		E	R †		E	R †		E	R †		E	R †	
interv-ariant mean Ct ± SD	11.5 ± 1.0	13.0 ± 0.7		15.3 ± 1.1	16.9 ± 1.1		19.2 ± 1.1	20.4 ± 1.0		22.5 ± 1.2	23.9 ± 1.0		26.0 ± 1.0	27.3 ± 0.7		29.5 ± 1.2	30.7 ± 1.0	
var. ^+^ lowest mean Ct var. ^+^ highest mean Ct	9.9 13.6	11.9 14.2		13.2 17.0	15.1 18.5		17.2 20.8	18.8 21.8		20.3 24.1	22.3 25.4		24.2 27.5	26.2 28.2		27.2 31.1	28.7 32.2	
inter-variant CV [%]	8.7	5.4		7.2	6.5		5.7	4.9		5.3	4.2		3.8	2.6		4.1	3.3	
MagNA + LM *	E	R †		E	R †		E	R †		E	R †		E	R †		E	R †	
inter-variant mean Ct ± SD	12.4 ± 1.2	13.6 ± 1.0		16.0 ± 0.8	17.3 ± 0.8		19.6 ± 1.2	21.1 ± 1.1		23.2 ± 1.1	24.5 ± 1.2		26.7 ± 1.1	28.1 ± 1.3		30.1 ± 1.2	31.3 ± 0.9	
var. ^+^ lowest mean Ct var. ^+^ highest mean Ct	10.2 14.9	11.9 15.2		14.6 17.5	16.2 18.7		18.1 22.3	20.1 23.6		22.3 26.1	23.6 27.4		25.8 29.3	26.6 31.0		28.4 32.1	30.0 33.1	
inter-variant CV [%]	9.7	7.4		5.0	4.6		6.1	5.2		4.7	4.9		4.1	4.6		4.0	2.9	
NX-48 + LM *	E	R †		E	R †		E	R †		E	R †		E	R †		E	R †	
inter-variant mean Ct ± SD	13.4 ± 1.3	14.6 ± 0.9		16.4 ± 0.8	17.5 ± 0.7		20.0 ± 1.0	21.3 ± 0.9		23.8 ± 1.1	25.0 ± 1.0		27.3 ± 1.3	28.7 ± 1.2		30.5 ± 1.5	31.9 ± 1.4	
var. ^+^ lowest mean Ct var. ^+^ highest mean Ct	10.9 15.1	12.8 15.6		14.5 17.6	16.1 18.4		17.9 21.3	19.5 22.5		21.7 25.2	23.5 26.5		25.2 29.2	27.1 30.5		28.1 32.9	29.9 34.1	
inter-variant CV [%]	9.7	6.2		4.9	4.0		5.0	4.2		4.6	4.0		4.8	4.2		4.9	4.4	

†—RdRP, ‡—ORF1ab, ^+^—individual variant, *—LightMix.

**Table 2:** Comparison of PCR efficiencies [%] of all assessed diagnostic approaches for 10 clinically relevant SARS-CoV-2 genomic variants. Efficiency (%) = ((10e (−1/slope)) − 1) × 100).

		B.1 [%]	B.1.258.17 [%]	A.27 [%]	Alpha [%]	Beta [%]	Gamma [%]	Delta [%]	Eta [%]	Iota [%]	Omicron [%]
Alinity	N/RdRP	95.3	97.1	95.6	95.5	92.0	89.1	96.0	92.5	81.6	104.8
cobas	ORF1ab	98.8	99.5	103.2	101.9	105.0	100.1	104.3	103.0	99.7	104.0
	E	95.9	95.8	100.4	100.2	102.4	96.8	102.6	101.7	95.8	106.8
STARLet	E	99.9	94.7	91.1	93.8	92.2	88.9	101.0	94.7	96.6	105.6
	S/RdRP	87.1	88.2	84.5	84.3	86.0	81.1	88.0	81.5	89.1	96.5
	N	85.1	94.0	92.0	94.6	87.1	80.6	87.3	88.3	91.8	104.6
Maelstrom + LM *	E	97.6	83.3	87.6	90.6	90.7	83.6	92.7	87.8	93.5	97.4
	RdRP	94.4	88.7	91.6	90.3	100.3	88.1	95.0	89.0	91.4	101.4
MagNA + LM *	E	90.4	92.6	96.6	93.6	98.6	87.6	82.6	87.3	90.3	100.3
	RdRP	89.1	91.9	98.2	93.8	96.5	89.1	80.1	87.8	85.4	102.4
NX-48 + LM *	E	93.3	83.6	99.1	97.3	95.1	88.3	93.9	91.4	95.8	104.2
	RdRP	89.7	82.7	100.1	97.0	93.9	88.1	91.4	90.5	86.5	106.4
	mean	93.1	91.0	94.5	94.4	95.0	88.5	92.9	91.3	91.5	102.9
	SD	4.7	5.7	5.7	4.7	5.9	5.6	7.6	6.1	5.2	3.4
	minimal	85.1	82.7	84.5	84.3	86.0	80.6	80.1	81.5	81.6	96.5
	maximal	99.9	99.5	103.2	101.9	105.0	100.1	104.3	103.0	99.7	106.8

* LightMix.

**Table 3:** Inter-test comparison of RdRP gene mean Ct values with standard deviations (SD) and coefficient of variation (CV) generated by all diagnostic approaches tested and difference between platform with highest (hi) and lowest (lo) individual test mean Ct value (Δ hi-lo) for respective concentration of 10 clinically relevant SARS-CoV-2 genomic variants altogether and separately.

		1 × 10^4^ [PFU/mL]	1 × 10^3^ [PFU/mL]	1 × 10^2^ [PFU/mL]	1 × 10^1^ [PFU/mL]	1 × 10^0^ [PFU/mL]	1 × 10^−1^ [PFU/mL]
all variants	inter-assay mean Ct ± SD	13.5 ± 1.5	16.9 ± 1.5	20.6 ± 1.6	24.2 ± 1.6	27.6 ± 1.7	30.9 ± 1.8
	Δ hi-lo	5.7	6.5	6.6	6.8	7.2	7.6
	inter-assay CV [%]	11.1	8.9	7.8	6.6	6.2	5.8
B.1	inter-assay mean Ct ± SD	13.3 ± 1.8	16.1 ± 1.8	19.9 ± 1.8	23.7 ± 1.8	27.2 ± 2.1	30.4 ± 2.0
	Δ hi-lo	5.0	5.5	5.3	4.9	6.0	5.1
	inter-assay CV [%]	13.5	11.2	9.0	7.6	7.7	6.6
B.1.258.17	inter-assay mean Ct ± SD	14.0 ± 1.7	17.4 ± 1.7	21.2 ± 2.0	24.9 ± 1.7	28.1 ± 2.1	31.7 ± 2.4
	Δ hi-lo	4.8	5.0	5.9	4.9	5.4	6.7
	inter-assay CV [%]	12.1	9.8	9.4	6.8	7.5	7.6
A.27	inter-assay mean Ct ± SD	13.5 ± 1.5	16.5 ± 1.7	20.4 ± 1.8	23.5 ± 1.7	27.3 ± 1.7	30.5 ± 1.9
	Δ hi-lo	4.6	4.7	5.0	4.7	5.1	5.2
	inter-assay CV [%]	11.1	10.3	8.8	7.2	6.2	6.2
Alpha	inter-assay mean Ct ± SD	13.9 ± 1.3	17.3 ± 1.4	21.0 ± 1.6	24.5 ± 1.7	27.9 ± 1.6	31.2 ± 1.8
	Δ hi-lo	3.8	4.3	5.1	5.3	4.9	5.7
	inter-assay CV [%]	9.4	8.1	7.6	6.9	5.7	5.8
Beta	inter-assay mean Ct ± SD	13.4 ± 1.2	17.2 ± 1.4	20.4 ± 1.3	23.9 ± 1.3	27.3 ± 1.3	30.7 ± 1.3
	Δ hi-lo	3.1	3.9	3.5	4.2	3.8	3.9
	inter-assay CV [%]	9.0	8.1	6.4	5.4	4.8	4.2
Gamma	inter-assay mean Ct ± SD	13.2 ± 1.5	17.0 ± 1.0	20.5 ± 1.3	24.0 ± 1.1	27.7 ± 1.6	31.5 ± 1.7
	Δ hi-lo	4.2	2.6	3.5	3.3	4.8	5.0
	inter-assay CV [%]	11.4	5.9	6.3	4.6	5.8	5.4
Delta	inter-assay mean Ct ± SD	12.2 ± 0.9	15.5 ± 1.1	19.4 ± 1.3	22.9 ± 1.4	26.5 ± 1.4	29.6 ± 1.5
	Δ hi-lo	2.4	2.8	3.6	3.8	4.0	4.0
	inter-assay CV [%]	7.4	7.1	6.7	6.1	5.3	5.1
Eta	inter-assay mean Ct ± SD	13.2 ± 1.6	16.6 ± 1.5	20.7 ± 1.6	24.5 ± 1.7	28.1 ± 1.9	30.8 ± 1.1
	Δ hi-lo	4.4	4.5	4.9	4.6	5.2	3.3
	inter-assay CV [%]	12.1	9.0	7.7	6.9	6.8	3.6
Iota	inter-assay mean Ct ± SD	14.7 ± 1.3	17.9 ± 1.4	22.1 ± 1.3	25.8 ± 1.3	29.0 ± 1.2	32.7 ± 1.1
	Δ hi-lo	3.5	3.6	3.7	4.0	3.2	3.0
	inter-assay CV [%]	8.8	7.8	5.9	5.0	4.1	3.4
Omicron	inter-assay mean Ct ± SD	13.5 ± 1.4	17.2 ± 1.7	20.8 ± 1.7	24.0 ± 1.5	27.0 ± 1.8	29.8 ± 1.7
	Δ hi-lo	4.0	4.9	5.4	4.8	5.6	5.1
	inter-assay CV [%]	10.4	9.9	8.2	6.3	6.7	5.7

## Data Availability

Additional data presented in this study are available on request from the corresponding author.

## Supplementary Materials

The following supporting information can be downloaded at: https://www.mdpi.com/article/10.3390/pathogens11040462/s1. Table S1: STARLet’s mean Ct values obtained by testing serial dilutions of 10 SARS-CoV-2 genomic variants; Figure S1: Assessment of systemic error, robustness of the systems used, and pipetting accuracy.
