# Supplementary material for: Comparative Evaluation of Six SARS-CoV-2 Real-Time RT-PCR Diagnostic Approaches Shows Substantial Genomic Variant–Dependent Intra- and Inter-Test Variability, Poor Interchangeability of Cycle Threshold and Complementary Turn-Around Times
Source: Pathogens. 2022 Apr 12;11(4):462. doi: 10.3390/pathogens11040462 (PMC9029830; doi:10.3390/pathogens11040462)
Supplement: Supplementary file 1 [file pathogens-11-00462-s001.zip › pathogens-1645616-supplementary.pdf]

Supplementary Table S1. STARLet's mean Ct values obtained by testing serial dilutions of 10 SARS-CoV-2 genomic variants

	virus concentration [PFU/mL]																	
genomic variant	1×10^4			1×10^3			1×10^2			1×10^1			1×10^0			1×10^{-1}		
target	E	S/RdRp	N	E	S/RdRp	N	E	S/RdRp	N	E	S/RdRp	N	E	S/RdRp	N	E	S/RdRp	N
B.1 (D614G)	14.7	13.3	12.5	16.9	16.7	17.0	20.8	20.6	20.8	24.2	24.3	24.4	27.9	28.0	28.0	30.7	31.5	31.4
B.1.258.17	16.4	15.9	16.1	19.7	19.4	19.3	23.7	23.7	23.4	26.8	26.9	25.9	29.7	30.1	29.0	34.0	34.3	34.1
A.27	15.3	14.4	15.3	18.3	17.5	18.4	22.9	22.6	23.0	25.9	25.5	25.6	29.9	29.8	29.7	32.6	32.7	32.7
Alpha	15.5	15.5	15.7	19.2	19.0	19.4	22.3	23.1	22.5	26.7	27.0	26.4	29.2	30.4	29.2	33.0	34.3	33.2
Beta	15.0	14.2	18.9	18.8	18.0	22.6	21.8	21.3	25.8	26.2	25.8	30.0	28.9	28.6	32.8	32.7	32.9	37.7
Gamma	15.5	13.9	13.4	18.5	17.9	18.2	22.0	21.6	21.8	25.2	25.2	25.3	28.7	28.2	28.5	34.0	34.2	33.8
Delta	13.4	12.9	11.3	15.9	16.1	16.0	19.5	20.7	19.7	23.5	24.4	23.2	26.5	27.5	26.6	29.4	30.9	29.9
Eta	15.9	15.4	18.8	19.0	18.8	21.9	23.6	23.5	26.4	26.8	26.8	29.8	30.2	30.8	33.1	33.0	32.4	35.3
Iota	15.8	15.0	19.7	19.6	18.6	23.4	23.2	22.3	26.9	26.8	26.2	30.6	28.7	28.9	33.0	33.5	33.4	37.9
Omicron	15.5	14.9	14.6	19.1	18.9	18.1	22.8	23.0	21.7	25.9	26.3	24.9	28.9	29.5	27.9	31.3	31.8	30.6

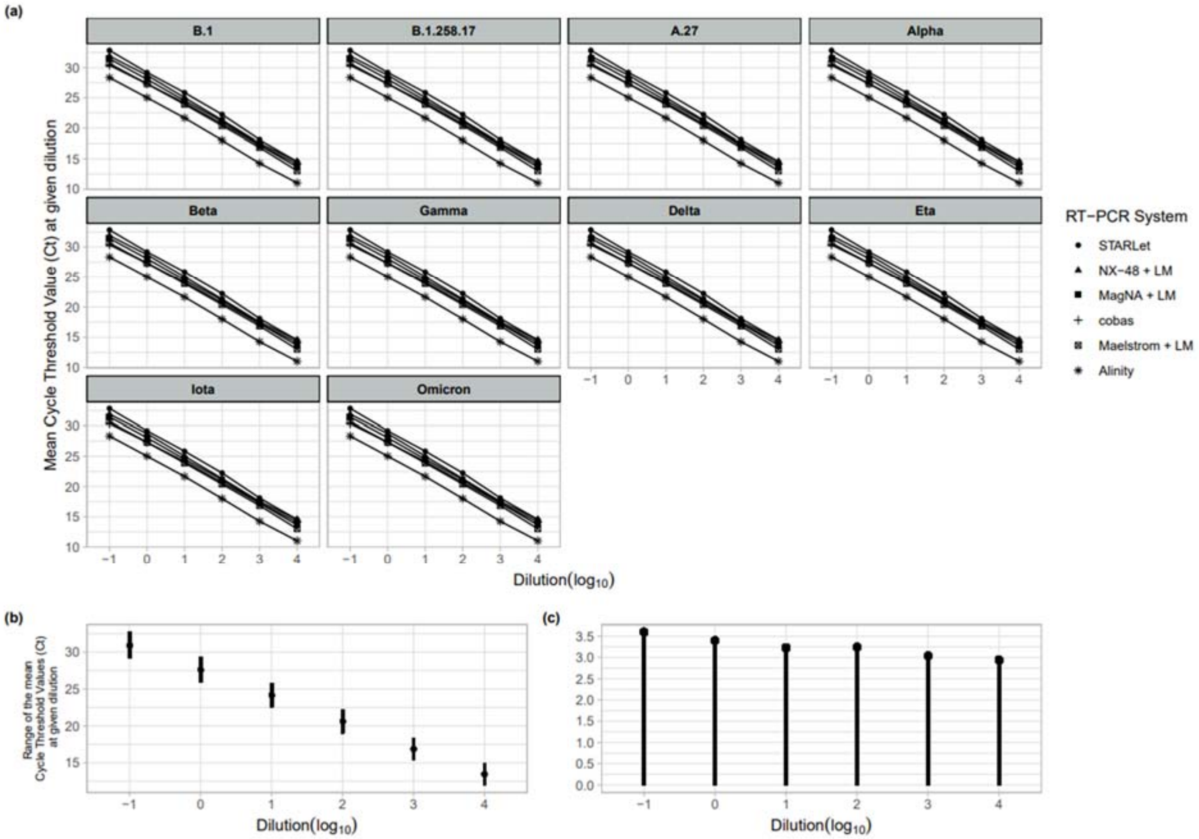

Supplementary Figure S1. Assessment of systemic error, robustness of the systems used, and pipetting accuracy. (a) The boxplot of each variant shows the distribution and respective means of the Ct values across entire range of dilutions in order to assess the level of systemic error present in the study. It is shown, that the ordering of systems, seen in Figure 1 does not change at any dilution point, indicating correct sample processing and pipetting. In other words, the level of dilution does not affect the ordering of mean Ct values across systems; (b) Further investigation of this property reveals almost constant range of mean Ct values across all systems; (c) Absolute Ct values of each range reveal only slight variation (it drops from 3.5 at the lowest dilution point to 3.0 at the highest dilution point). This characteristic is indicative of the robustness of the systems used and accurate pipetting across all dilutions.
